# Old age and depression in Ghana: assessing and addressing diagnosis and treatment gaps

**DOI:** 10.1080/16549716.2019.1678282

**Published:** 2019-11-04

**Authors:** Peter Lloyd-Sherlock, Sutapa Agrawal, Mary Amoakoh-Coleman, Selasie Adom, Ebenezer Adjetey-Sorsey, Ilaria Rocco, Nadia Minicuci

**Affiliations:** a School of International Development, University of East Anglia, Norwich, UK; b Public Health Foundation of India, New Delhi, India; c Department of Epidemiology, Noguchi Memorial Institute for Medical Research, University of Ghana, Legon, Ghana; d Department of Psychiatry, Korle-bu Teaching Hospital, Accra, Ghana; e HelpAge Ghana, Accra, Ghana; f Neuroscience Institute, CNR, Padova, Italy

**Keywords:** Depression, prevalence, feasibility study, older adults, mental health, Africa

## Abstract

**Background**: There is limited evidence about the prevalence of depression among older people in sub-Saharan Africa, about access to treatment or the potential efficacy of community-based interventions.

**Objective**: Using nationally representative data from the WHO SAGE survey, we examine the prevalence of and factors associated with depression among people aged 50 and over in Ghana. Compare self-reported diagnosis and a symptom algorithm to assess treatment gaps and factors associated with the size of gap. Assess the feasibility of a small community-based intervention specifically for older people.

**Method**: Prevalence and treatment data were taken from the WHO SAGE 2007 survey in Ghana, including 4,725 people aged 50 or over. Outcomes of interest were self-reported depression and diagnosis of depression derived from a symptom-based algorithm. The data were subjected to bivariate and multivariate analysis. In parallel, a pilot intervention was conducted with 35 older people, which included screening by a trained psychiatrist and follow-up group sessions of psychotherapy.

**Results**: The symptomatic algorithm reported an overall rate of 9.2 per cent for the study population, with associations with female sex and older age. The treatment gap for these cases was found to be 83.0 per cent. The implementation of the pilot study was perceived as effective and replicable by stakeholders and there was some evidence of enhanced outcomes for people with mild depression.

**Conclusions**: Large numbers of older people in Ghana experience depression, but very few have access to treatment. There is an urgent need to develop and validate community-based services for older people experiencing this condition.

## Background

There is growing recognition of the important contribution made by mental health to the global burden of disease and quality of life in low and middle-income countries [1]. At the same time, it is acknowledged that most mental health conditions, including depression receive a low policy priority relative to other areas of health [2]. Compared to other world regions, research on depression and mental health in later life in sub-Saharan Africa remains especially scant. The estimated pooled prevalence of depressive symptoms among people aged 50 and over in 15 sub-Saharan African countries in 2002–3 was over 9 per cent, indicating that the condition is not uncommon in later life [3]. Studies from sub-Saharan Africa show strong associations between depression, poor functional status, poor quality of life and co-morbid conditions [4,5].

The limited available evidence suggests that there is a very large ‘treatment gap’ for people identified as affected by depression or other mental health disorders. Estimates indicate that less than one in ten people with depression in low-resource settings have access to treatment, and that levels of access are even lower for poorer socio-economic groups [6,7]. In part this reflects a high degree of policy neglect. By 2010 only 42 per cent of countries in sub-Saharan African had adopted an official mental health policy, with less than one per cent of the region’s total health budget allocated to mental health [6]. The bulk of this limited expenditure is allocated to psychiatric hospitals, with very little emphasis on the provision of mental health services through primary care facilities [6]. Consequently, large numbers of people with mental health conditions are unlikely to have access to mental health services and are therefore unlikely to be diagnosed or treated. These effects may be especially important for older adults, as depression among people at older ages often goes undiagnosed [8].

One way to gauge the extent of diagnosis and treatment gaps is to compare responses to a simple self-report screening question, such as whether respondents report they have been told by a doctor that they have depression, and more complex, symptom-based approaches. This paper analyses survey data on the prevalence of depression among adult people living in Ghana from WHO’s Study on Global Ageing and adult health (SAGE) conducted during 2007–10, which includes both self-reported depression and a symptom-based algorithm. The paper also analyses SAGE data on reported treatment for depression over a two week and 12 month period. Alongside this, the paper sets out and reviews the findings of an independent feasibility study based on applying the SAGE algorithm in the field and linking this to a consultation and referral provided by a trained psychiatrist, with support from a local NGO.

## Survey and methods

The comparison of self-reported and diagnosed depression is based on data from the WHO Study on Global Aging and Adult Health (SAGE). This includes a nationally representative household and individual survey conducted in Ghana in 2007. Sampling methods were based on the design developed for the World Health Survey [9] where a probability sampling design was employed using multi-stage, stratified, random cluster samples. The primary sampling units were stratified by region and location (urban/rural) and enumeration areas within each stratum were selected. Details are available at [10] and the SAGE website (www.who.int/healthinfo/systems/sage).

SAGE takes the same approach to measuring depression as most other international health surveys, such as the WHO World Health Survey and the World Mental Health Survey. A number of different symptom-based algorithms have been widely applied using standardised structured diagnostic interviews designed for assessment by trained lay interviewers. The Epidemiological Studies Depression Scale (CES-D-10) is a 10-item Likert scale questionnaire for assessing depressive symptoms in the past week [11]. It includes three items on depressed affect, five items on somatic symptoms, and two on positive affect. Another widely used symptomatic approach is the Geriatric Depression Scale (GDS) short form, which consists of a 15-item questionnaire with binary yes/no responses [12]. The Composite International Diagnostic Interview (CIDI), developed by the World Health Organisation (WHO), is a diagnostic tool, and applies a modified set of CIDI items. These items are specified in accordance with the a diagnosis of ‘depressive episode’ according to criteria in the International Statistical Classification of Diseases and Related Health Problems, 10th revision, Diagnostic Criteria for Research [13,14]. The items included in the CIDI algorithm are listed in Table 1. Versions of CIDI have been applied in a number of WHO surveys, including the World Mental Health Survey and SAGE.

**Table 1.** Items included in the WHO SAGE depression diagnostic algorithm.

During the last 12 months, have you had a period lasting several days when you felt sad, empty or depressed?
During the last 12 months, have you had a period lasting several days when you lost interest in most things you usually enjoy such as personal relationships, work or hobbies/recreation?
During the last 12 months, have you had a period lasting several days when you have been feeling your energy decreased or that you are tired all the time?
**INTERVIEWER: IF ANY ONE OF RESPONSES TO PREVIOUS QUESTIONS IS ‘YES’, CONTINUE**
Was this period [of sadness/loss of interest/low energy] for more than 2 weeks?
Was this period [of sadness/loss of interest/low energy] most of the day, nearly every day?
During this period, did you lose your appetite?
Did you notice any slowing down in your thinking?
Did you notice any problems falling asleep?
Did you notice any problems walking up too early?
During this period, did you have any difficulties concentrating; for example, listening to others, working, watching TV, listening to the radio?
Did you notice any slowing down in your moving around?
During this period, did you feel anxious and worried most days?
During this period, were you so restless or jittery nearly every day that you paced up and down and couldn’t sit still?
During this period, did you feel negative about yourself or like you had lost confidence?
Did you frequently feel hopeless-that there was no way to improve things?
During this period, did your interest in sex decrease?
Did you think of death, or wish you were dead?
During this period, did you ever try to end your life?

Interviews were conducted by trained enumerators, rather than relevant health professionals. Participants who had previously been diagnosed with depression and had been receiving treatment during the last 12 months were categorised as self-reported depressed. SAGE also applies the items that make up the CIDI symptomatic algorithm (Table 1). Our symptomatic depression items were based on the World Mental Health Survey version of the Composite International Diagnostic Interview [14]. The diagnosis of depression was based on the International Classification of Diseases, 10th revision (ICD-10), diagnostic criteria for research (DCR) for depressive episodes [13], which were derived from an algorithm that took into account respondents reporting symptoms of depression during the past 12 months [15]. Participants endorsed at least 4 of 10 depressive symptoms lasting 2 weeks most of the day or all of the day. According to the ICD–10–DCR criterion B, at least two of the following three symptoms needed to be present in order to be diagnosed as depressive: depressed mood, loss of interest, and fatigue [15]. Additionally, the respondents who responded positively to the question, ‘Have you been taking any medications or other treatment such as attending therapy or counselling sessions for depression during the last 12 months?’ were also added to the symptom-based depression [16].

The household wealth variable provided in the dataset followed the standard WHO approach to estimating permanent income from survey data on household ownership of durable goods, neighbourhood and dwelling characteristics, and access to water, sanitation, electricity etc [17]. An index score was calculated for each household by weighting each income indicator by the coefficient of the first principal component. All individuals present in the household were assigned the same score, and the score was categorized into quintiles such as poorest, poor, middle, rich and richest.

Standard descriptive statistics were calculated for all variables. Differences in categorical variables were tested using χ2 tests. The non-parametric Fisher exact test was used whenever the applicability condition for the chi-square was not verified. Multivariable logistic regression models were used to estimate the odds ratios (OR) of symptom-based depression, with age, gender, residence, marital status, educational status and wealth quintile as covariates, based on our previous knowledge of associations in other countries. In all data analysis, sampling weights were used to account for the complex, multistage design of the SAGE survey [10]. All analyses were conducted using STATA version 14 (StataCorp, College Station, Texas, USA).

SAGE received human subjects testing and ethics council approval from research review boards local to each participating country. In Ghana Ethical approval was provided by the University of Ghana Medical School Ethics and Protocol Review Committee. Written informed consent was obtained from each respondent prior to interview and examination. Our secondary analysis is based on SAGE de-identified data which is available in the WHO public domain and does not require further ethics committee approval.

## Results

Table 2 shows the self-reported and the symptoms-based prevalence for adults aged 50 and over. The self-report measure identified 67 cases of depression, compared to 395 identified by the symptom-based approach. This shows that the single self-report question missed the large majority (92.8%) of cases identified by the symptom-based algorithm, and indicates the scale of the treatment gap for this condition. For both, there were bivariate associations with age, sex and marital status.

**Table 2.** Distribution % [n] of characteristics WHO SAGE Ghana, 2007.

	Total sample (n=4725)	Self Reported	Chi-square p- value	Symptom-based	Chi-square p-value
Prevalence		1.6[67]		9.3[395]	
Age (years)			<0.0001***		<0.0001***1
[50-59]	1883	0.7[11]		7.0[117]	
[60-69]	1305	1.4[17]		9.9[118]	
[70-79]	1071	2.5[24]		10.6[103]	
[80+]	473	3.5[15]		13.4[57]	
Sex			<0.0001***		<0.0001***
Males	2379	0.9[20]		7.4[167]	
Females	2345	2.3[47]		11.3[228]	
Marital status			0.001**		<0.0001***
Never married	58	0.0[0]		14.0[7]	
Married/Cohabiting	2739	1.0[23]		7.2[174]	
Separated/Divorced	624	2.8[17]		11.2[68]	
Widowed	1276	2.3[27]		12.3[146]	
Education			0.063*		0.022*
None	2380	1.5[35]		10.3[244]	
Primary	894	2.5[22]		8.4[75]	
Secondary	878	0.9[8]		8.3[73]	
Higher	151	1.3[2]		4.0[6]	
Location			0.0578		0.314
Urban	1927	2.0[35]		9.8[172]	
Rural	2803	1.3[32]		8.9[226]	
Wealth quintile			0.343		0.019*
Poorest	939	0.8[7]		8.0[68]	
Q2	940	2.0[17]		12.1[102]	
Q3	949	1.5[13]		9.4[80]	
Q4	934	1.7[15]		9.3[81]	
Richest	961	1.7[15]		7.8[67]	

The other bivariate associations varied by approach. People aged 50 and over with no completed education had a higher symptomatic prevalence (10.3%) than those with some education, but this was not the case with self-report. A likely explanation is that more educated people were more aware of the condition or had better access to diagnostic services.

Table 3 presents ORs for symptom-based depression. It shows significantly increased risk of reporting depression for females (OR = 1.27 95%CI (1.01–1.82)), being aged 80 or over (OR = 1.92 95%CI (1.22–3.02)) and for the second-bottom wealth quintile (OR = 1.51 95%CI (1.04–2.19)). The low numbers of self-reported cases preclude similar multivariable analysis. Table 3 also presents ORs for people reporting depressive symptoms but who have not been diagnosed as depressed. This shows a strong negative association with having higher education: in other words, people who have symptoms of depression and have education to a higher level are more likely to have been diagnosed as depressed that those with lower levels of education.

**Table 3.** Odds ratio (with 95%CI) of symptom-based depression and diagnosis.

Characteristics	OR(95%CI) for reporting symptom-based depression	P values	OR (95%CI) for reporting symptoms of depression but not a diagnosis	P values
Age in years				
50–59	1.00		1.00	
60–69	1.28 (0.94–1.73)	0.115	1.23(0.89–1.70	0.206
70–79	1.27(0.91–1.74)	0.155	1.08(0.77–1.52)	0.766
80+	1.96(1.24–3.02)	0.004	1.26(0.79–2.01)	0.785
Sex				
Male	1.00		1.00	
Female	1. 35(1.01–1.82)	0.046	1.30(0.98–1.72)	0.068
Educational status				
None	1.00		1.00	
Primary	0.94(0.68–1.31)	0.733	0.83(0.58–1.18)	0.304
Secondary	0.89(0.62–1.28)	0.523	0.84(0.57–1.24)	0.377
Higher	0.65(0.20–2.09)	**0.472**	0.26(0.09–0.79)	**0.018**
Marital Status				
Never married	1.00		1.00	
Currently Married/cohabiting	0.76(0.32–1.83)	0.547	0.64(0.27–1.53)	0.316
Separated/divorced	1.08(0.44–2.64)	0.874	0.81(0.33–1.99)	0.644
Widowed	1.08(0.44–2.63)	0.869	0.94(0.39–2.27)	0.884
Location				
Urban	1.00		1.00	
Rural	0.86(0.66–1.12)	0.275	0.81(0.61–1.07)	0.644
Wealth quintile				
Poorest	1.00		1.00	
Q2	1.51(1.04–2.19)	0.027	1.22(0.84–1.75)	0.291
Q3	1.25(0.86–1.82)	0.246	1.15(0.78–1.70)	0.482
Q4	1.40(0.95–2.07)	0.087	1.19(0.79–1.80)	0.397
Richest	1.21(0.76–1.93)	0.423	1.20(0.75–1.93)	0.453

Table 4 shows numbers of people who were diagnosed with depression, as well as those reporting to have taken some form of treatment over the past year or past two weeks (as mutually exclusive responses). The small number of observations does not permit robust bivariate or multivariate analysis. Table 4 also shows the number of older people treated for depression who still reported depressive symptoms. All 27 respondents who had received treatment during the last year still reported symptoms according to the SAGE criteria. Of those 14 who had received treatment in the previous two weeks, only one was free of symptoms. It is possible that in some cases treatment may have reduced the severity of depression without eliminating it.

**Table 4.** SAGE respondents reporting diagnosis and treatment of depression.

	Diagnosed (% of all respondents and n)	Treated in last 2 weeks (% of diagnosed and n)	Treated in last 2 weeks but reporting symptoms (n)	Treated in last 12 months (% of diagnosed and n)	Treated in last 12 months but reporting symptoms (n)
Male	0.8[20]	25.0[5]	29.4[5]	50.0[10]	58.8[10]
Female	2.3[47]	19.2[9]	24.2[8]	36.2[17]	51.5[17]
Urban	2.0[35]	20.0[7]	30.4[7]	37.1[13]	56.5[13]
Rural	1.3[32]	21.9[7]	22.2[6]	43.8[14]	51.9[14]
Total	67	20.9[14]	13	40.3[27]	27

## Feasibility study

This study sought to apply the SAGE screening algorithm in a community setting and link this to a diagnostic consultation and, where needed, follow-up referral with a trained health professional. The validation study was conducted in Accra in Ghana among older people who attended a day centre run by the non-governmental organization, HelpAge Ghana. The centre provides educational, recreational and social activities for older people and additionally offered regular health screening services and check-ups. Attendees come from different neighbourhoods of Accra and their involvement is facilitated by a network of community volunteers.

A pre-study orientation meeting was held at the day centre to provide information about the study and address issues of fear of potential stigmatization due to the condition being studied. An information sheet about the proposed study was also provided. We then recruited 35 people, aged 50 years and above, without any cognitive impairment as per our screening protocol, (appendix 1), using convenience sampling. We targeted a female to male ratio of 60:40% due to the documented higher female depression prevalence ratios [18]. Full agreement and ethical approval for the study to proceed was received by HelpAge Ghana and the Ghana Health Service Ethics Review Committee.

All the older people attended the initial orientation meeting and were screened using WHO’s Mini-Mental tool [19], which was adapted to context. This involved assessment of their ability to do word recognition, awareness and word recall using a thirteen item scale. Cognitive impairment was defined by a score of 10 or below. Thirty-one people scored above 10. Those 26 older people with cognitive impairment were excluded from the rest of the study. These were further interviewed by the professional psychiatrist (to assess the nature of their condition), and provided with information about their condition and a referral letter, as appropriate.

Thirty five older people (12 males and 23 females) were selected and assessed using items of the WHO SAGE questionnaire relating to depression (appendix 2) using a trained enumerator. Table 5 presents selected characteristics of the study participants. Lastly, on another day, each participant was independently evaluated by a trained psychiatrist who assessed whether or not they are depressed and the severity of any depression using routine clinical practice guidelines in assessing depression. These clinical evaluations were not recorded, but the psychiatrist made detailed written notes for later analysis. In cases of depression or mood or psychosocial problems, participants went through weekly sessions of psychotherapy for one month, and eventually those were still having depression provided with information about their condition by the psychiatrist and with a referral letter, as appropriate. English, Twi and Ga languages were used for interviews. The trained enumerator and the professional psychiatrist could speak both Twi and Ga. Older people who were not able to communicate in any of these languages were excluded. Data on the SAGE items were entered into SPSS version 20. Analysis was conducted through individual case reviews and comparison of performance on the two assessments. Proportions of agreement and divergence were estimated.

**Table 5.** Selected characteristics of study participants.

		60–69 years old	70–79 years old	80+ years old
Sex	Male	3	6	3
	Female	10	7	6
Marital status	Single	0	3	0
	Married	4	2	3
	Separated	1	3	1
	Widowed	8	5	5
Pension	Yes	6	10	5
	No	7	3	4
Total		13	13	9

Table 6 compares the results of using the SAGE measurement tool and the psychiatric evaluations across the 35 study participants. For 30 out of 35 participants, the diagnosis of depression corresponded across both approaches: of those, 10 were identified as depressed. In five cases the two approaches produced differing results. All of these five cases were identified as depressed by the SAGE tool, but not by the psychiatric evaluations. The psychiatric evaluation identified low mood in all these discordant cases, along with other depressive symptoms, and in two cases recommended further psychiatric intervention. However, the combination and severity of these symptoms was not enough to justify a formal diagnosis of depression according to the International Classification of Diseases [13].

**Table 6.** Comparison of depression evaluations from SAGE and psychiatric evaluation.

	N	%
Not Depressed (Both)	20	57.1
Depressed SAGE/Not Depressed Psyc	5	14.3
Not Depressed SAGE/Depressed Psyc	0	0.0
Depressed (Both)	10	28.6
Agreement	30	85.7
Discordant	5	14.3
Total	35	
Sensitivity of SAGE tool		10/10 = 100.0%
Specificity of SAGE tool		20/25 = 80.0%

Of the 35 cases, none reported having had a previous diagnosis for depression, and none were receiving treatment for depression either at the time of the intervention or in the past. Following the intervention, one case of severe depression, which had not been previously diagnosed, was referred to the city’s only psychiatric hospital. Additionally, five follow-on group therapies were provided to all participants by a trained psychiatrist over a three week period. There is some evidence that these group sessions led to improved symptoms for mild cases of depression. For example, all participants who had been screened positively for depression by SAGE were rescreened after the intervention. Of these, none were found to be clinically depressed, although reported three mild mood disturbances. According to the therapist’s notes:

Significant among these was the case of the severely depressed woman in her late seventies. She was never married and had no children. She reported that she felt disrespected by people around her, including the children of her siblings. For several years, she had been affected by a visual impairment requiring surgery, but had been unable to access this service. In the early stages of the intervention, it was uncertain whether she would benefit from the group approach and so I considered working with her on an individual basis. However, in the later sessions, she reported examples of practical changes in her thinking patterns and that her interpersonal relationships had improved as a result of the intervention.

There was unanimous agreement among the participants that the intervention had been beneficial for their mood, sleep and general wellbeing. All expressed a wish for the sessions to continue beyond the intervention and to be extended to other older people, but this was not possible due to a lack of funding.

## Discussion

An association between symptomatic depression and female sex is consistently reported by other studies [20,21]. Our reported association with older age does not coincide with findings in some high-income countries, where prevalence has been reported to fall between middle and later adulthood [22]. However, several studies set in low and middle-income countries do report associations with older ages [20,23]. For example, a systematic review of prevalence studies in Ethiopia found consistent associations with older ages [24].

In terms of overall prevalence of symptomatic depression, the 9.3 per cent rate reported by the SAGE symptomatic algorithm for Ghana broadly conforms to findings of some other studies in sub-Saharan Africa [3,7,25]. Our results are also broadly consistent with those of Thapa, Martinez and Clausen [20] who report a prevalence of 6.7 per cent for mild depression in Ghana, using the same 2007 SAGE survey data. By contrast, a study of 262 people aged 65 and over conducted in Ghana’s Volta Region applies the GDS and reports a prevalence of 37.8 per cent [26]. The authors of that study speculate that this high prevalence may have been caused by non-specified local effects, which may not apply equally across Ghana as a whole.

Studies of older adults in other sub-Saharan African countries also report divergent prevalences. In South Africa a national SAGE study using CES-D reported the overall prevalence of mild depression at only 2.7 per cent [20]. However, data from a separate national study using CES-D-10 found a prevalence of 33.1 per cent for adults aged 65 and over [27]. A study based on a shortened version of the SAGE instrument administered to South Africans aged 50 and over who were either HIV-infected or lived with another relative with HIV reported 42.4 per cent had reported a depressive episode in the previous year [28]. In that survey, questionnaires were administered by professional nurses, rather than enumerators. This suggests that variations across these surveys may in part be due to the sensitivity of symptomatic diagnosis to the form of questioning and the capacity of interviewers.

Cross-national and cross-cultural comparisons of depression prevalence outside sub-Saharan Africa also show large variations. For example, WHO’s World Mental Health Survey, which applied CIDI to 18 countries, reported 12 month prevalences ranging from 2.2 per cent in Japan to 18.4 per cent in Brazil [22]. It is speculated that these variations may in part be caused by the challenges of developing measurement tools that are valid in different cultural and linguistic settings [29]. Sweetland et al (2014:230) observe that:

‘an instrument used satisfactorily in one African setting may or may not have the same applicability in another setting, or with a different population, even within the same country.’ [30]

This calls for further studies to adapt instruments to local settings and to assess how the experience of the enumerator and other aspects of the interview context may influence reporting of depressive symptoms [31,32]. Nonetheless, the close association between the psychiatric evaluation and the SAGE algorithm in the intervention study indicates that the SAGE tool is effective at capturing this condition, at least in settings like urban Accra.

Our reported diagnosis gap was 83.0 per cent of people with symptomatic depression and our treatment gaps (96.5 per cent for two weeks; 89.9 per cent for either two weeks or 12 months) fall within the range of other similar studies in the region for various mental health disorders. National estimates range from 75 per cent in South Africa [33] to over 90 per cent in Ethiopia [34]. There are a number of studies on depression treatment gap in different sub-Saharan African settings, but these focus on younger age groups and conditions such as postpartum and perinatal depression [35,36].

Beyond the potential effects of enumerator capacity, there are numerous other possible limitations on the robustness of our findings. If rates of symptomatic depression are under-reported, for the reasons discussed above, the actual diagnosis gap may be even larger. The high level of conformity between the SAGE tool and the independent psychiatric evaluation suggests that this is not a major problem. However, it is possible that under-reporting is more frequent in the field where individual enumerator capacities and the interview setting are not conducive to capturing sensitive data. Conversely, it is possible that size of the diagnosis gap may be exaggerated if participant recall of being diagnosed for depression is unreliable or if the diagnosis was not clearly explained by the health professional. Studies outside sub-Saharan Africa shows that self-reporting depression can be significantly affected by a range of factors, including denial, stigma, current mood and memory [37–39].

In the face of limited diagnostic and biometric data in Ghana and much of sub-Saharan Africa, the use of self-report responses on conditions like depression is understandable [40]. However, the gap in diagnosis is so great that these surveys are likely to be very misleading, in terms of understating both overall prevalence and specific rates for groups less able to access diagnostic services.

The few other studies of treatment gap in LMICs often exclude people at older ages [41]. The SAGE data show a very large gap between the number of older Ghanaians reporting depressive symptoms and the numbers who had been diagnosed or were being treated. Of the 395 cases, only 14 reported receiving any treatment in the previous two weeks and 27 in the past year. SAGE does not ask about specific forms of treatment, and therefore this figure may overstate the number of people who were receiving appropriate treatment. The small number of respondents who were being treated does not permit for a robust assessment of treatment efficacy. Nonetheless, it is noteworthy that only one of 41 people treated was assessed as not depressed at the time of the survey.

The feasibility study has a number of limitations. The number of participants was small and they were all recruited from urban neighbourhoods of the capital city, Accra. As such, the results are not generalizable to older people in all parts of Ghana where health services are often less available. Despite this, none of the 35 participants, including those identified as depressed by SAGE, had ever been diagnosed or treated. The limited data from the group sessions indicate this form of therapy may have some benefits for milder cases of depression. There is therefore a need for similar intervention studies elsewhere in Ghana and across sub-Saharan Africa.

The scale of the diagnosis and treatment gap accords with the limited available information on service availability in Ghana. As well as a general neglect of mental health issues, there are indications that depression in later life is particularly over-looked. Although programmes such as WHO’s MH Gap have made some progress in training primary health care workers about symptoms and treatment of conditions like depression, this does not make specific reference to geriatric depression [42]. The higher prevalence of co-morbidities among this population is likely to encourage a focus on somatic symptoms rather than underlying psychiatric causes and to explain depressive symptoms simply in terms of old age. As such, if depressed older people do not present at one of the country’s three hospitals offering specialist psychiatric care, their chances of diagnosis are very slim. Some relevant forms of medication are covered by Ghana’s National Health Insurance Scheme, which includes almost all people over the age of 60. However, drugs for many conditions, especially depression, are often unavailable free of charge, representing a further barrier to treatment for older people in a country where most receive no pension [43]. More generally, knowledge of depression among Ghanaians remains limited and the condition is stigmatised, especially among the older generations.

Health policy for older people represents a new and neglected issue for sub-Saharan Africa [44]. The same is true for mental health services. It is not surprising, then, that the mental health of older people is especially over-looked and that access to diagnosis and effective treatment is almost entirely lacking. Ghana’s current state provision of mental health services for older people is largely focussed on a single psychiatric ward for around 35 older people dealing with a wide range of conditions. Yet older Ghanaians are making increasing use of health services, with people aged 50 and over accounting for 18 per cent of outpatient consultations in 2016 [45]. High rates of treatment-seeking represent both an incentive and an opportunity for enhancing awareness of geriatric depression among front-line primary health workers across all parts of the country.

## Conclusion

This study contributes to the limited current knowledge about depression in later life in sub-Saharan Africa. Using a symptom-based algorithm, it shows that 9.3 per cent of participants aged 50 and over were depressed. This equates to a total population of 229,000 in 2010 [46]. Of these only 7.2 per cent had been diagnosed and only 3.5 per cent had received any form of treatment during the two weeks prior to the survey. The small feasibility study indicates the potential for interventions in community settings to mitigate depressive symptoms among older adults.

## Paper context

Research on depression among older adults in sub-Saharan Africa is very limited. This paper seeks to address this knowledge gap by analysing data from a WHO survey of older people’s health conducted in Ghana. We find that around one in ten people aged 50 or more experienced depression and that almost none had access to effective treatment. The paper also assesses the feasibility and potential impact of s simple, community-based intervention to improve treatment.
